# Gitelman Syndrome Presenting With Syncope and Treatment‐Refractory Hypokalemia in A Young Woman: A Case Report

**DOI:** 10.1002/ccr3.72272

**Published:** 2026-03-09

**Authors:** Iyassu S. Melkie, Abenezer A. Wolde, Lulit Y. Mengesha, Rahwa A. Kinfe, Chernet T. Mengistie, Zenebwork Y. Gubai

**Affiliations:** ^1^ School of Medicine, College of Health Sciences Addis Ababa University Addis Ababa Ethiopia; ^2^ School of Medicine, College of Health Sciences Gondar University Gondar Ethiopia

**Keywords:** Gitelman syndrome, hypokalemia, refractory electrolyte imbalance, salt‐wasting tubulopathy, SLC12A3 mutation

## Abstract

Gitelman syndrome (GS) is a rare autosomal recessive tubulopathy characterized by hypokalemic metabolic alkalosis, hypomagnesemia, and hypocalciuria. A 27‐year‐old woman presented with a witnessed syncopal episode, progressive weakness, and nausea. She reported a 3‐year history of muscle cramps, paresthesias, salt craving, and nocturia, with only transient correction of hypokalemia despite supplementation. Examination showed orthostatic hypotension and proximal muscle weakness, and ECG revealed flattened T and prominent U waves. Laboratory tests demonstrated severe hypokalemia (2.7 mmol/L), metabolic alkalosis, hypomagnesemia, renal potassium wasting, hypocalciuria, elevated renin and aldosterone, and a negative diuretic screen, consistent with GS. Severe hypokalemia is arrhythmogenic; ECG changes and syncope in this patient prompted monitored cardiac care and urgent correction. She was treated with intravenous and oral potassium and magnesium plus amiloride, leading to symptomatic improvement but persistently low‐normal potassium levels (3.3–3.7 mmol/L). Genetic testing confirmed a pathogenic *SLC12A3* variant. This case underscores the importance of considering GS in young adults with unexplained hypokalemia and the difficulty of achieving full biochemical correction despite optimal therapy.

## Key Clinical Message

Gitelman syndrome should be considered in young adults with unexplained hypokalemia, metabolic alkalosis, and hypomagnesemia. Despite appropriate potassium and magnesium replacement, complete biochemical correction is often difficult to achieve, highlighting the chronic and treatment‐refractory nature of this inherited tubulopathy.

## 1 Introduction

Gitelman syndrome (GS) is an autosomal recessive salt‐wasting renal tubulopathy characterized biochemically by hypokalemic metabolic alkalosis, hypomagnesemia, hypocalciuria, and secondary hyperaldosteronism [1]. It is rare, with an estimated prevalence of ~1 in 40,000 individuals [2]. GS is caused by bi‐allelic loss‐of‐function mutations in the SLC12A3 gene on chromosome 16q13, which encodes the thiazide‐sensitive Na^+–Cl^– cotransporter (NCC) in the distal convoluted tubule [3, 4]. Inactivating NCC mutations impair renal NaCl reabsorption, leading to mild volume depletion, chronic stimulation of the renin–angiotensin–aldosterone system, and renal K^+ and Mg^2+ wasting [1, 5].

Patients with GS typically present in late childhood, adolescence, or early adulthood [6]. Clinical manifestations are variable. Many patients are asymptomatic or only mildly symptomatic, often being diagnosed incidentally on routine blood tests [4, 6]. When present, symptoms reflect electrolyte depletion and volume contraction, including salt craving, fatigue, muscle weakness or cramps, paresthesias, tetany, and polyuria/nocturia [2, 4, 5]. Patients may have low or normal blood pressure due to chronic salt wasting [7]. Importantly, the chronic hypokalemia of GS frequently prolongs ventricular repolarization. While marked arrhythmias are uncommon [8], QT prolongation is often seen, and rare cases of syncope or sudden cardiac arrest have been reported [2, 8].

Diagnosis of GS relies on characteristic biochemical findings together with exclusion of other causes of hypokalemic alkalosis. Key laboratory features include persistent hypokalemia, metabolic alkalosis, hypomagnesemia, and low urinary calcium excretion [1, 4]. Plasma renin and aldosterone are typically elevated in the setting of normotension [7]. A formal urine diuretic screen and urinary electrolyte indices help exclude surreptitious diuretic use [9]. Genetic testing of SLC12A3 can confirm the diagnosis in most cases [1, 5].

Management of GS centers on liberalizing salt intake and aggressive electrolyte supplementation [9, 10]. Lifelong high‐dose oral potassium and magnesium are required, often in combination with potassium‐sparing diuretics such as amiloride or spironolactone to reduce renal K^+ losses [11]. Patients are encouraged to eat a high‐sodium, high‐potassium, and high‐magnesium diet [10]. Careful cardiac monitoring is recommended when hypokalemia is severe [12]. With treatment, many patients achieve near‐normal electrolytes; however, partial hypokalemia often persists due to renal losses [4, 9, 11].

We report a case of GS in a young woman who presented with syncope and severely refractory hypokalemia to highlight the clinical features, diagnostic considerations, and treatment challenges of this disorder.

## 2 Clinical History/Examination

A 27‐year‐old Ethiopian woman presented to the emergency department after a witnessed syncopal episode and 48 h of progressive generalized weakness, nausea, and two episodes of near‐syncope. She reported a three‐year history of intermittent muscle cramps, distal paresthesia, longstanding salt craving, polyuria, and nocturia (typically awakening twice nightly), and multiple prior outpatient courses of oral potassium replacement (40–80 mmol/day) that produced only transient correction. She denied vomiting, diarrheal illness, laxative use, prescribed or over‐the‐counter diuretics, herbal remedies, or illicit drug use. Her past medical history was otherwise unremarkable. Family history was notable for a maternal uncle described as having “similar cramps” treated with potassium tablets but without a formal diagnosis; there was no known consanguinity.

**FIGURE 1:**
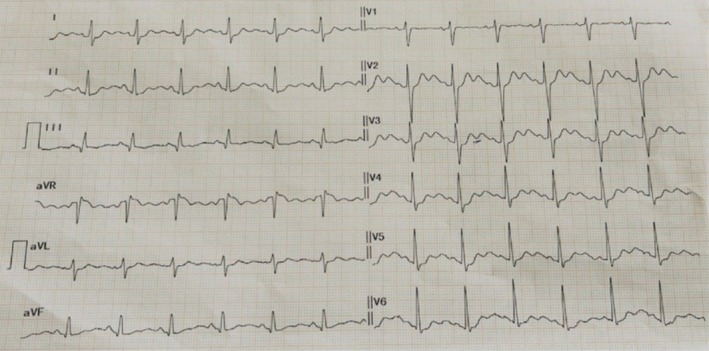
Electrocardiogram on admission showing sinus rhythm with flattened T waves, prominent U waves, and borderline QT prolongation, consistent with hypokalemia.

On examination, she was thin (BMI 19.8 kg/m^2^), alert and oriented, afebrile, pulse 88/min, and blood pressure 100/68 mmHg supine with a 12 mmHg orthostatic fall in systolic pressure on standing and a mild compensatory tachycardia. Cardiorespiratory and abdominal examinations were unremarkable. Neurological examination demonstrated proximal greater than distal weakness (hip flexion and shoulder abduction 4/5 bilaterally; distal strength 4+/5), intact sensation, and normal deep tendon reflexes; gait was mildly unsteady on heel walking, attributable to weakness. Admission 12‐lead ECG showed sinus rhythm ~90/min with flattened T waves, prominent U waves, and a borderline prolonged QTc (Figure 1).

## 3 Differential Diagnosis, Investigations, and Treatment

Initial laboratory testing confirmed marked hypokalemia (serum K^+^ 2.7 mmol/L) with metabolic alkalosis (serum HCO_3_^−^ 33 mmol/L), hypomagnesemia (serum Mg^2+^ 0.55 mmol/L), normal serum sodium, and preserved renal function (serum creatinine 0.7 mg/dL). Spot urine testing and a timed collection demonstrated renal potassium wasting (24‐h urinary K^+^ 65 mmol/24 h) and hypocalciuria (spot urine Ca/Cr ratio 0.04). Plasma renin activity (6.5 ng/mL/h) and aldosterone (37 ng/dL) were both elevated in the context of normal/low blood pressure, consistent with a secondary hyperreninemic state. A formal urine diuretic screen was performed during admission and returned negative (Table 1), supporting endogenous renal salt‐wasting rather than surreptitious diuretic use. Thyroid function and random cortisol were within reference ranges. Based on the combination of hypokalemia, metabolic alkalosis, hypomagnesemia, hypocalciuria, elevated renin and aldosterone, normal blood pressure, and a negative diuretic screen, a **clinical diagnosis of Gitelman syndrome** was made.

**TABLE 1:** Laboratory investigations.

Laboratory/investigation	At admission	After 24 h	At discharge (day 5)	8‐week follow‐up	Reference range
Hemoglobin	13.2 g/dL	—	13.1 g/dL	13.0 g/dL	12.0–16.0 g/dL
White blood cells	6.5 × 10^9^/L	—	6.3 × 10^9^/L	6.4 × 10^9^/L	4.0–11.0 × 10^9^/L
Platelets	250 × 10^9^/L	—	248 × 10^9^/L	255 × 10^9^/L	150–400 × 10^9^/L
MCV	88 fL	—	87 fL	88 fL	80–100 fL
Sodium (Na^+^)	137 mmol/L	136 mmol/L	137 mmol/L	137 mmol/L	135–145 mmol/L
Potassium (K^+^)	2.7 mmol/L	3.2 mmol/L	3.4 mmol/L	3.3–3.7 mmol/L	3.5–5.0 mmol/L
Chloride (Cl^−^)	96 mmol/L	98 mmol/L	97 mmol/L	98 mmol/L	98–107 mmol/L
Bicarbonate (HCO_3_^−^)	33 mmol/L	30 mmol/L	28 mmol/L	26–28 mmol/L	22–28 mmol/L
Creatinine	0.7 mg/dL	0.7 mg/dL	0.7 mg/dL	0.7 mg/dL	0.6–1.1 mg/dL
Magnesium (Mg^2+^)	0.55 mmol/L	0.75 mmol/L	0.85 mmol/L	0.90 mmol/L	0.70–1.10 mmol/L
Calcium (total, corrected)	9.1 mg/dL	9.0 mg/dL	9.0 mg/dL	9.1 mg/dL	8.5–10.2 mg/dL
Phosphate	1.0 mmol/L	—	1.1 mmol/L	1.0 mmol/L	0.8–1.5 mmol/L
AST	22 U/L	—	20 U/L	21 U/L	0–40 U/L
ALT	18 U/L	—	17 U/L	18 U/L	0–40 U/L
ALP	68 U/L	—	65 U/L	66 U/L	40–120 U/L
Total bilirubin	0.7 mg/dL	—	0.7 mg/dL	0.7 mg/dL	0.1–1.2 mg/dL
TSH	1.8 mIU/L	—	1.7 mIU/L	1.8 mIU/L	0.4–4.0 mIU/L
Free T4	1.1 ng/dL	—	1.1 ng/dL	1.1 ng/dL	0.8–1.8 ng/dL
INR	1.0	—	1.0	1.0	0.9–1.2
aPTT	32 s	—	31 s	32 s	25–40 s
Arterial blood gas (room air)	pH 7.48, pCO_2_ 41 mmHg, HCO_3_^−^ 33 mmol/L	pH 7.44, pCO_2_ 40 mmHg	pH 7.40, HCO_3_^−^ 28 mmol/L	pH 7.38, HCO_3_^−^ 26 mmol/L	pH 7.35–7.45
Urinalysis	Specific gravity 1.010; no protein; no blood; no glucose	—	—	—	—
24‐h urinary potassium	65 mmol/24 h	—	—	—	Elevated in renal wasting
Spot urine Ca/Cr ratio	0.04 (low)	—	—	—	Low in hypocalciuria
Plasma renin activity	6.5 ng/mL/h	—	—	—	0.2–2.8 ng/mL/h
Plasma aldosterone	37 ng/dL	—	—	—	4–31 ng/dL
Urine diuretic screen	Negative (performed during admission)	—	—	—	—

Because of symptomatic and ECG‐documented severe hypokalemia, she was admitted to a monitored bed and received continuous cardiac monitoring. Acute management included intravenous potassium chloride administered in divided doses (total 120 mmol over 24 h) and intravenous magnesium sulfate 2 g over 4 h with serial electrolyte monitoring; serum potassium rose to 3.2 mmol/L and magnesium to 0.75 mmol/L with partial symptomatic improvement. For ongoing management, she was commenced on oral magnesium oxide 400 mg three times daily, sustained‐release potassium chloride 40 mmol twice daily, and amiloride (initiated 5 mg daily and titrated to 10 mg daily) to reduce renal potassium wasting. She received counseling to moderately liberalize dietary sodium, increase potassium‐ and magnesium‐rich foods, and avoid substances known to exacerbate kaliuresis (excessive caffeine, licorice). No adverse events occurred during replacement, and renal function remained stable. The ECG abnormalities resolved, and the patient reported partial symptomatic improvement; therefore, no additional potassium‐sparing agent (e.g., spironolactone) or NSAIDs were initiated at discharge.

## 4 Outcome and Follow‐Up

The patient was discharged on hospital day five with serum potassium 3.4 mmol/L and magnesium 0.85 mmol/L and arranged for close outpatient follow‐up. Over eight weeks of outpatient care, adherence to oral supplements and amiloride produced notable improvement in cramps, fatigue, and presyncopal symptoms, with no further syncopal episodes, allowing return to usual activities. However, serum potassium remained low‐normal to borderline (3.3–3.7 mmol/L), consistent with the chronic biochemical pattern of Gitelman syndrome.

Genetic testing at follow‐up was performed using a targeted NGS tubulopathy panel (mean coverage ~150×, > 98% of targets ≥ 20×). Two compound heterozygous pathogenic variants were identified in SLC12A3: c.179C> T (p.Thr60Met) in exon 1, and c.2221G> A (p.Gly741Arg) in exon 18. Both variants were confirmed by Sanger sequencing. The laboratory classified each as pathogenic according to ACMG criteria (e.g., PM1, PM2, PP3). Both variants are rare in gnomAD (allele frequencies ~0.0001–0.0003) and are reported in ClinVar. No copy‐number alterations were detected. Parental segregation testing was not performed. This confirmed the diagnosis of Gitelman syndrome. Limitations of the inpatient workup were therefore addressed: the negative urine diuretic screen obtained during admission helped exclude surreptitious diuretic use, and genetic confirmation was obtained subsequently through the outsourced test.

## 5 Discussion

This patient's presentation illustrates the key features and management challenges of GS. Persistent refractory hypokalemia is a hallmark of GS. In fact, chronic hypokalemia is “the main finding” in affected patients [6]. Despite aggressive replacement, most GS patients remain mildly hypokalemic, requiring ongoing supplementation. In the long term, GS is a lifelong condition: serum K^+ and Mg^2+ typically improve with treatment but rarely normalize completely [6, 9]. There are limited prospective data on the longitudinal course, but existing reports indicate that GS does not resolve with age, and symptom burden (fatigue, cramps) often persists into adulthood [4, 13].

Differentiating GS from other causes of hypokalemic alkalosis is critical. In this case, exclusion of Bartter syndrome and diuretic abuse was important. Compared with GS, classic Bartter syndromes present much earlier (often antenatally or in infancy) and commonly feature hypercalciuria [5, 6, 10, 14]. In contrast, GS patients typically present later with hypocalciuria and normal/low blood pressure [14]. Measurement of 24‐h urinary calcium can help distinguish GS (low urinary Ca) from Bartter (high urinary Ca) [5, 14]. Surreptitious diuretic use must also be considered in unexplained hypokalemia. A comprehensive diuretic screen and the context of chronic symptoms help exclude this; in our patient, a negative diuretic screen supported a renal tubular etiology [9].

Therapeutically, GS often requires a multi‐pronged approach. The cornerstone is high‐dose electrolyte supplementation. Oral potassium chloride and magnesium oxide were used in our patient as recommended [9, 13]. Adjunctive therapies aim to reduce renal potassium wasting. Potassium‐sparing diuretics such as amiloride or spironolactone counteract hyperaldosteronism and have been shown to improve serum K^+ in GS [10, 11]. Indeed, antialdosterone therapy (spironolactone and amiloride) has been reported to significantly raise serum K^+ and reduce urinary K^+ excretion in GS patients [11]. In one series, combined spironolactone/amiloride treatment raised mean serum K^+ from ~2.6 to 3.4 mmol/L (*p* < 0.001) and reduced renal K^+ clearance [15]. Amiloride and spironolactone are both reasonable; the choice depends on patient tolerance and side effect profiles [10, 15]. In our patient, amiloride was initiated together with oral magnesium oxide and sustained‐release potassium chloride after acute intravenous replacement; serum K rose to 3.2 mmol/L and Mg to 0.75 mmol/L, and the ECG abnormalities normalized. Because symptoms and ECG changes improved while on amiloride, we judged that escalation to an additional potassium‐sparing agent was not indicated at discharge.

Dietary counseling (including liberal salt intake, high‐potassium foods, and avoidance of licorice or excessive caffeine) is also advised [14, 16]. In refractory cases, adjunctive therapies (e.g., prostaglandin synthesis inhibitors) are sometimes considered, though their benefit is clearer in Bartter variants than in GS [11, 17]. COX inhibitors (most commonly indomethacin) can raise serum potassium in some series and may be considered for selected refractory Gitelman cases, but indomethacin was the most effective in small studies and is limited by gastrointestinal intolerance and reductions in eGFR; therefore, NSAIDs should be reserved for refractory patients after nephrology review and monitoring [11].

Regarding outcomes, GS is generally considered to have a benign long‐term prognosis [9]. Life expectancy is usually normal, and most patients avoid life‐threatening complications with treatment [4]. However, quality of life can be significantly affected by chronic symptoms. For example, a large GS cohort found fatigue and muscle cramps in over 80% of patients [13], and many report salt craving and nocturia. Even with treatment, GS patients often report ongoing tiredness and reduced well‐being, underscoring the chronicity of the condition [4, 9, 13]. Persistent hypokalemia can prolong the QT interval; although serious ventricular arrhythmias are uncommon, vigilance is warranted [8]. Indeed, isolated cases of syncope and sudden cardiac arrest have been attributed to untreated GS [2, 8]. GS has also been associated with metabolic perturbations: recent studies suggest carriers of SLC12A3 mutations may have altered glucose metabolism, and GS patients appear to have a higher incidence of type 2 diabetes compared with controls [18]. There are even reports of extrarenal features such as chondrocalcinosis in some GS patients, likely related to chronic hypomagnesemia [19]. Renal function is typically preserved, but rare cases of chronic kidney disease have been reported, possibly secondary to long‐term hypokalemia and hypovolemia [4, 10].

Gitelman syndrome shows marked genotype–phenotype heterogeneity: different SLC12A3 variants produce variable biochemical profiles (including magnesium levels), age at onset, and occasional extrarenal associations; precise genotyping therefore aids prognostication and comparison with reported cases [1, 20]. Detailed variant reporting (c./p. notation, exon and zygosity) enables nuanced genotype–phenotype comparisons, for example, Koca et al. documented an atypical presentation of GS with autoimmune thyroiditis [20].

Our therapeutic goals in this patient are sustained symptom control, prevention of arrhythmia, and maintenance of near‐normal potassium and magnesium levels. She will continue oral supplementation and amiloride with periodic electrolyte and ECG monitoring. Escalation to spironolactone or NSAIDs would be considered only if symptoms recur or biochemical control becomes inadequate. In summary, this case of GS highlights the importance of suspecting GS in young patients with unexplained hypokalemic metabolic alkalosis. Its management requires aggressive and multidisciplinary therapy. While GS often carries a favorable prognosis in terms of mortality, the burden of chronic electrolyte management and symptomatology is nontrivial. Ongoing follow‐up is essential to monitor electrolytes, growth (if pediatric), blood pressure, and ECG changes.

## 6 Conclusion

Gitelman syndrome is a lifelong salt‐wasting tubulopathy that often presents in young adults with muscle cramps, salt craving, and refractory hypokalemia. Our patient's syncope was precipitated by severe hypokalemia due to GS. This case reinforces that clinicians should suspect GS when unexplained hypokalemia is accompanied by metabolic alkalosis, hypomagnesemia, and hypocalciuria in a normotensive patient. Early recognition allows genetic confirmation and the institution of tailored therapy. Despite maximal treatment with potassium and magnesium supplements and potassium‐sparing agents, many GS patients continue to exhibit low‐normal serum potassium. Nonetheless, aggressive electrolyte management can alleviate symptoms, reduce arrhythmic risk, and improve quality of life in this rare disorder.

## Author Contributions

**Iyassu S. Melkie:** conceptualization, visualization, writing – original draft. **Abenezer A. Wolde:** visualization, writing – review and editing. **Lulit Y. Mengesha:** data curation, resources. **Rahwa A. Kinfe:** data curation, resources. **Chernet T. Mengistie:** writing – original draft, writing – review and editing. **Zenebwork Y. Gubai:** supervision, writing – review and editing.

## Funding

The authors has nothing to report.

## Ethics Statement

IRB review and approval were waived for this case report.

## Consent

Written informed consent was obtained from the patient for publication of the case details and accompanying images.

## Conflicts of Interest

The authors declare no conflicts of interest.

## Data Availability

The data underlying the results presented in this work are available within the manuscript.

## References

[1] N. Li and H. F. Gu , “Genetic and Biological Effects of SLC12A3, a Sodium‐Chloride Cotransporter, in Gitelman Syndrome and Diabetic Kidney Disease,” Frontiers in Genetics 13 (2022): 799224. 35591852 10.3389/fgene.2022.799224 PMC9111839

[2] A. Kondo , C. Nagano , S. Ishiko , et al., “Examination of the Predicted Prevalence of Gitelman Syndrome by Ethnicity Based on Genome Databases,” Scientific Reports 11, no. 1 (2021): 16099. 34373523 10.1038/s41598-021-95521-6 PMC8352941

[3] K. Nozu , T. Yamamura , T. Horinouchi , et al., “Inherited Salt‐Losing Tubulopathy: An Old Condition but a New Category of Tubulopathy,” Pediatrics International 62, no. 4 (2020): 428–437. 31830341 10.1111/ped.14089

[4] J. Fujimura , K. Nozu , T. Yamamura , et al., “Clinical and Genetic Characteristics in Patients With Gitelman Syndrome,” Kidney Int Rep 4, no. 1 (2019): 119–125. 30596175 10.1016/j.ekir.2018.09.015 PMC6308995

[5] K. P. Schlingmann and J. H. F. De Baaij , “The Genetic Spectrum of Gitelman(−Like) Syndromes,” Current Opinion in Nephrology and Hypertension 31, no. 5 (2022): 508–515. 35894287 10.1097/MNH.0000000000000818 PMC9415222

[6] D. Mamalis , T. Stratigou , N. Vallianou , G. Ioannidis , and T. Apostolou , “Persistent Hypokalemia due to a Rare Mutation in Gitelman's Syndrome,” Saudi Journal of Kidney Diseases and Transplantation 31, no. 1 (2020): 259. 32129221 10.4103/1319-2442.279949

[7] W. Ji , J. N. Foo , B. J. O'Roak , et al., “Rare Independent Mutations in Renal Salt Handling Genes Contribute to Blood Pressure Variation,” Nature Genetics 40, no. 5 (2008): 592–599. 18391953 10.1038/ng.118 PMC3766631

[8] A. Bettinelli , C. Tosetto , G. Colussi , G. Tommasini , A. Edefonti , and M. G. Bianchetti , “Electrocardiogram With Prolonged QT Interval in Gitelman Disease,” Kidney International 62, no. 2 (2002): 580–584. 12110021 10.1046/j.1523-1755.2002.00467.x

[9] S. O. Mohammadi , A. Shafiee , A. Bolds , R. Siripurapu , and S. Kankanala , “Managing Gitelman Syndrome: Socioeconomic Barriers and Clinical Outcomes,” Kidney Dial 5, no. 2 (2025): 21.

[10] A. Blanchard , D. Bockenhauer , D. Bolignano , et al., “Gitelman Syndrome: Consensus and Guidance From a Kidney Disease: Improving Global Outcomes (KDIGO) Controversies Conference,” Kidney International 91, no. 1 (2017): 24–33. 28003083 10.1016/j.kint.2016.09.046

[11] A. Blanchard , R. Vargas‐Poussou , M. Vallet , et al., “Indomethacin, Amiloride, or Eplerenone for Treating Hypokalemia in Gitelman Syndrome,” Journal of the American Society of Nephrology 26, no. 2 (2015): 468–475. 25012174 10.1681/ASN.2014030293 PMC4310664

[12] A. Bezzeccheri , G. Di Giovanni , M. Belli , et al., “The Impact of Gitelman Syndrome on Cardiovascular Disease: From Physiopathology to Clinical Management,” Reviews in Cardiovascular Medicine 23, no. 8 (2022): 289. 39076641 10.31083/j.rcm2308289 PMC11266949

[13] D. N. Cruz , A. J. Shaer , M. J. Bia , R. P. Lifton , and D. B. Simon , “Gitelman's Syndrome Revisited: An Evaluation of Symptoms and Health‐Related Quality of Life,” Kidney International 59, no. 2 (2001): 710–717. 11168953 10.1046/j.1523-1755.2001.059002710.x

[14] E. Wan , R. J. Unwin , and S. B. Walsh , “Liquorice, Liddle, Bartter or Gitelman—How to Differentiate?,” Nephrology, Dialysis, Transplantation 34, no. 1 (2019): 38–39. 10.1093/ndt/gfy199 29982819

[15] G. Colussi , G. Rombol&agrave , M. E. De Ferrari , M. Macaluso , and L. Minetti , “Correction of Hypokalemia With Antialdosterone Therapy in Gitelman and Rsquo;s Syndrome,” American Journal of Nephrology 14, no. 2 (1994): 127–135. 8080005 10.1159/000168701

[16] F. Francini , L. Gobbi , V. Ravarotto , et al., “The Dietary Approach to the Treatment of the Rare Genetic Tubulopathies Gitelman's and Bartter's Syndromes,” Nutrients 13, no. 9 (2021): 2960. 34578838 10.3390/nu13092960 PMC8467039

[17] X. Peng , C. Chen , J. Tu , Y. Lin , H. Li , and H. Geng , “Long‐Term Indomethacin Treatment in a Chinese Child With Gitelman Syndrome: Case Report and Literature Review on Its Efficacy and Tolerance,” American Journal of Case Reports [Internet] 24 (2023): e941627, 10.12659/AJCR.941627. 38069462 PMC10720922

[18] A. Blanchard , M. Vallet , L. Dubourg , et al., “Resistance to Insulin in Patients With Gitelman Syndrome and a Subtle Intermediate Phenotype in Heterozygous Carriers: A Cross‐Sectional Study,” J Am Soc Nephrol 30, no. 8 (2019): 1534–1545. 31285285 10.1681/ASN.2019010031 PMC6683723

[19] Z. Iqbal and J. A. Sayer , “Chondrocalcinosis and Gitelman Syndrome,” QJM 109, no. 8 (2016): 563–564. 27026693 10.1093/qjmed/hcw045 PMC4986430

[20] O. Koca , M. T. Alay , A. Murt , A. Kalayci Yigin , M. Seven , and I. Bavunoglu , “A Novel Homozygous SLC12A3 Mutation Causing Gitelman Syndrome With Co‐Existent Autoimmune Thyroiditis: A Case Report and Review of the Literature,” CEN Case Reports 13, no. 5 (2024): 330–338. 38308744 10.1007/s13730-023-00845-z PMC11442957

